# Late-Onset Focal Epilepsy: Electroclinical Features and Prognostic Role of Leukoaraiosis

**DOI:** 10.3389/fneur.2022.828493

**Published:** 2022-02-23

**Authors:** Elena Tartara, Elisa Micalizzi, Sofia Scanziani, Elena Ballante, Matteo Paoletti, Carlo Andrea Galimberti

**Affiliations:** ^1^Epilepsy Center, Istituto di Ricovero e Cura a Carattere Scientifico (IRCCS) Mondino Foundation, Pavia, Italy; ^2^Department of Brain and Behavioral Sciences, University of Pavia, Pavia, Italy; ^3^BioData Center, Istituto di Ricovero e Cura a Carattere Scientifico (IRCCS) Mondino Foundation, Pavia, Italy; ^4^Department of Mathematics, University of Pavia, Pavia, Italy; ^5^Department of Neuroradiology, Istituto di Ricovero e Cura a Carattere Scientifico (IRCCS) Mondino Foundation, Pavia, Italy

**Keywords:** epilepsy, elderly, interictal epileptiform discharges, leukoaraiosis, sleep

## Abstract

The aim of this study was to describe the electroclinical and prognostic characteristics, and to investigate the role of leukoaraiosis in outpatients with new-onset elderly focal epilepsy aged ≥60 years, referred to a tertiary epilepsy center between 2005 and December 31, 2020. Among the 720 patients who were referred to the center, we retrospectively selected 162 consecutive outpatients, with a first referral for recent-onset focal epilepsy of unknown cause (UC) or structural cause (SC), and collected a clinical and standard-Electroencephalogram (S-EEG), 24-h ambulatory EEG (A-EEG), and neuroimaging data. We also analyzed the seizure prognosis after titration of the first antiseizure medication (ASM). One hundred and four UC and 58 SC patients, followed up for 5.8 ± 5.3 years (mean ± SD), were included. Compared with the SC group, the patients with UC showed a predominance of focal seizures with impaired awareness (51.9% of cases) and focal to bilateral tonic-clonic seizures during sleep (25%); conversely, the SC group, more frequently, had focal to bilateral tonic-clonic seizures during wakefulness (39.6%) and focal aware seizures (25.8%) (*p* < 0.0001). Oral or gestural automatisms were prevalent in UC epilepsy (20.2 vs. 6.9% in the SC group, *p* = 0.04). In UC compared to patients with SC, interictal epileptiform discharges showed a preferential temporal lobe localization (*p* = 0.0007), low expression on S-EEG, and marked activation during deep Non-Rapid Eye Movement (NREM) sleep (*p* = 0.003). An overall good treatment response was found in the whole sample, with a probability of seizure freedom of 68.9% for 1 year. The cumulative probability of seizure freedom was significantly higher in the UC compared with the SC group (*p* < 0.0001). The prognosis was worsened by leukoaraiosis (*p* = 0.012). In the late-onset focal epilepsy of unknown cause, electroclinical findings suggest a temporal lobe origin of the seizures. This group showed a better prognosis compared with the patients with structural epilepsy. Leukoaraiosis, *per se*, negatively impacted on seizure prognosis.

## Introduction

Focal epilepsy in the elderly is attracting an increasing attention in the literature due to its acknowledged high prevalence and the diagnostic and therapeutic challenges it presents. These may be due to its peculiar clinical characteristics, high frequency of comorbidities, and low rate of interictal epileptiform abnormalities on wake EEG (increasing significantly in sleep) (1–4).

The published data on epilepsy in the elderly mainly refer to structural forms, which are most frequently observed in hospital inpatient settings (5, 6). Over 30% of newly diagnosed focal epilepsy in the elderly, particularly in outpatient settings, is of unknown etiology (1).

Several published studies suggest that epilepsy in the elderly carries a good outcome, but factors affecting its prognosis are poorly known. The patients with structural focal epilepsy may have a different prognosis *vs*. the cases with epilepsy of an unknown cause, but data on this are conflicting (7–10).

The purpose of this study is to characterize a new-onset focal epilepsy in elderly outpatients. To this end, our sample was divided into etiological groups, i.e., those with epilepsy of an unknown cause and those with symptomatic epilepsy (a structural etiology), and was evaluated from different perspectives: clinical, EEG and prognostic (11).

In addition, we explored the possible etiological and prognostic role of leukoaraiosis (white matter abnormalities usually related to cerebrovascular risk factors, frequently encountered on neuroimaging in elderly people). Although leukoaraiosis has not traditionally been considered an etiological factor in this setting, a recent experimental research highlighted the possible role of “occult cerebrovascular disease” as a possible trigger of epileptogenesis through a cascade of events (such as microangiopathy, blood-brain-barrier dysfunctions, and inflammation) that could affect the brain circuit functioning (12).

So far, data on the association of this imaging marker with a late-onset epilepsy and interpretations of its meaning have been controversial (13–16).

## Materials and Methods

This study was conducted in individuals aged 60 years or over, with newly diagnosed unprovoked epileptic seizures of unknown cause or of structural etiology, who were referred to a tertiary epilepsy center, and it aimed primarily to:

- analyze the clinical characteristics of focal epileptic seizures at the time of their first presentation;
- evaluate the yield of an extensive EEG assessment at the time of diagnosis, characterizing the pattern of occurrence of interictal epileptiform discharges (IEDs) during wake and sleep EEG recordings;
- define the effectiveness of the antiseizure medications (ASM).

Secondary aims were to estimate the factors that are possibly associated with seizure recurrence, and to investigate the possible role of leukoaraiosis.

Leukoaraiosis was defined as the presence of brain CT/MRI white matter hypoxic-ischemic abnormalities (17, 18).

Among the 2,816 patients who were consecutively referred to the outpatient practice of a single senior epileptologist (C.A.G.) at the Epilepsy Center of the IRCCS Mondino Foundation from January 1, 2005 to December 31, 2020, 720 were aged ≥60 years at the time of referral. Within this elderly cohort, we retrospectively selected 162 patients meeting the following criteria:

- a first referral for recent onset of suspected focal epileptic seizures; and
- a firm diagnosis, maintained over a minimum of 1 year of follow-up, of epilepsy of an unknown cause (UC) or a structural cause (SC), based on clinical history, general and neurological examination, EEG findings and neuroimaging studies, and has never been previously treated with ASM.

Exclusion criteria were the presence of dementia, psychogenic seizures, or rapidly growing cerebral tumors (high grade glial brain tumor or atypical meningioma).

Leukoaraiosis and non-focal cortical atrophy were not considered etiological factors.

The following data were collected:

- demographics;
- seizure semeiology (19);
- age at first seizure;
- age at first neurological referral;
- duration of follow-up (from the first to the last visit);
- general and neurological comorbidities;
- type and adverse effects of ASM prescribed at diagnosis; and
- duration of seizure freedom after reaching the first ASM target dose.

Moreover, the findings of the following investigations, performed in most patients at the time of the diagnosis, were also collected:

- standard EEG (S-EEG);
- 24-h ambulatory EEG (A-EEG), including whole-night sleep EEG recording and/or laboratory EEG-polysomnography (PSG) during a spontaneous afternoon nap; and
- neuroradiological assessment with brain MRI or CT (including evaluation of presumable causes of epilepsy and leukoaraiosis assessment, through Fazekas scale) (17, 18).

### EEG Analysis

The S-EEGs were classified on the basis of the possible presence of interictal epileptiform discharges (IEDs). The A-EEG and PSG recordings were reviewed in order to detect the possible occurrence of IEDs, and determine their relationship with different vigilance conditions (wakefulness; sleep stages scored by the standard criteria), and their localization and lateralization (20). In the presence of several IEDs *foci*, preferential localization of the most active focus was considered for the analysis.

### Statistics

Statistical analyses were performed using R 4.0.2. Descriptive statistics, which presented the continuous variables as mean, standard deviation, and median, categorical variables as row counts and percentage. The level of significance was set at 5%.

The comparison between patients with UC and with SC was performed using Wilcoxon test for independent samples for numeric variables, while Chi-squared test was used to compare categorical variables.

The probability of achieving a seizure freedom was described through Kaplan-Meier survival function, complemented by 95% CIs calculated through a log transformation of Survival function as suggested in (21). The censored observations were identified when no seizure was observed at the last observation. The difference between the survival functions of the two groups was tested through a Log-rank test.

The multivariate analysis on the covariates that affects the probability of seizure freedom was performed through Cox proportional hazards model.

## Results

### Demographic and Clinical Characteristics

The 162 patients included had a follow-up (mean ± S.D.) of 5.8 ± 5.3 years (median 4.3 years). Epilepsy was defined as UC in 104 of them (64.2%) and as SC in 58 (35.8%).

Table 1 presents the demographic, clinical, EEG, and neuroimaging data of these two groups of patients.

**Table 1:** Demographic, clinical, EEG, and neuroimaging characteristics of the whole sample grouped by focal epilepsy syndrome.

**Variables**	**Unknown** **cause (UC)** ***N* = 104**	**Structural** **cause (SC)** ***N* = 58**
**Socio-demographic and clinical**		
Gender (female), *n (%)*	49 (47.1)	24 (41.4)
**Age at first seizure, years**		
Mean ± SD Median	70.5 ± 6.7 70.5	70.9 ± 8.3 69.1
**Age at first referral, years**		
Mean ± SD Median	73.2 ± 7.2 73.0	74.2 ± 7.2 74.7
**Seizure types**		
Single unprovoked seizure, *n (%)*	6 (5.7)	4 (6.8)
Focal with impaired awareness*, n (%)*	17 (42.5)	9 (30.0)
Focal to bilateral tonic-clonic during wakefulness *n (%)*	11 (27.5)	14 (46.6)
Focal aware onset*, n (%)*	5 (12.5)	4 (13.3)
Focal to bilateral tonic-clonic during sleep, *n (%)*	6 (15)	3 (10)
Cardiovascular risk factors, *n (%)* - Arterial hypertension - Dyslipidemia - Atrial fibrillation - Smoke - Diabetes - Carotid atheromasia	63 (60.6) 19 (18.2) 19 (18.2) 19 (18.3) 4 (3.8) 6 (5.8)	42 (72.4) 14 (24.1) 20 (34.5) 10 (17.2) 9 (15.5) 11 (18.9)
EEG		
S-EEG, *n (%)* IED presence, *n (%)*	104 (100) 13 (12.5)	58 (100) 10 (17.2)
IED localization (EEG derivations), *n (%)*		
Frontal Temporal Parietal Occipital	0 13 (100) 0 0	1 (10) 5 (50) 4 (40) 0
A-EEG, *n (%)* IEDs in wakefulness, *n (%)*	87 (83.6) 24 (27.6)	33 (56.9) 14 (42.4)
Localization of IEDs on EEG derivations		
Temporal Frontal Occipital	24 (100) 0 0	7 (50) 7 (50) 0
IEDs in sleep, *n (%)*	71 (72.4)	27 (27.5)
Most frequent localization		
Temporal derivations Frontal/frontal central derivations Occipital derivations Exclusively 1-2 NREM Exclusively 3-4 NREM Activation in NREM 3-4 All NREM stages REM, *n (%)*	47 (67.1) 23 (32.8) 0 8 (9.2) 21 (24.1) 19 (21.8) 22 (25.3) 1 (1.1)	7 (26.9) 17 (65.4) 2 (7.7) 8 (20.5) 4 (10.2) 5 (5.1) 12 (30.8) 0
**Neuroimaging**		
CT, *n (%)*	10 (9.6)	9 (15.5)
MRI, *n (%)*	94 (90.4)	49 (84.5)
Leukoaraiosis, *n (%)* Non-focal cortical atrophy, *n (%)* Leukoaraiosis + atrophy, *n (%)*	51 (49) 1 (0.9) 18 (17.3)	36 (62) 4 (6.9) 10 (17.2)
**Fazekas scale score**		
Mean/median	2.34/2	2.95/3
Number of concomitant medications >3,		
*N of patient (%)*	42 (40.8)	10 (90.9)

*S-EEG, standard EEG recording; and A-EEG, 24-h ambulatory EEG recording*.

The main systemic comorbidities in the whole population were the cardiovascular risk factors, the most frequent being arterial hypertension (found in 60.6% of the patients with UC and 72.4% of the patients with SC), without statistical differences between the two groups. Diabetes, dyslipidemia, and atrial fibrillation were significantly more present in patients with SC (respectively, *p* = 0.02, *p* = 0.01, *p* = 0.03). The patients with UC took a median of 3 medications other than ASM, while patients with SC took a significantly higher number of drugs (median of 6) (*p* = 0.0002).

The most frequent structural etiologies were acute ischemic stroke (18.5% of the whole sample) and cerebral hemorrhage (6.8%). Other etiologies were neurosurgical outcomes, mesial temporal sclerosis (each documented in 3.1% of cases), meningioma (2.5%), subarachnoid hemorrhage (1.2%), and subdural hematoma (0.6%). The cerebral lesion was located in the left hemisphere in most cases, most frequently in the temporal-parietal-occipital regions (*n* = 27, 46.5%); less commonly, lesions were located in a single lobe (frontal *n* = 17, 29.3%, or temporal *n* = 13, 24.1%).

Of the 37 patients in whose etiology was a previous acute brain insult (mean ± S.D. age at the event 68.1 ± 7.4 years; median 65.4), six had shown acute provoked seizures (22). The mean time interval between the acute brain event and the first unprovoked seizure was 4.2 ± 5.1 years (mean ± S.D.; median 2.2 years).

### Seizure Types

Table 1 details the seizure types in the two groups.

Seizure types differed significantly between patients with UC and with SC (*p* < 0.0001). The UC group, compared with the SC group, was characterized by a predominance of focal seizures with impaired awareness and by a larger proportion of patients with focal to bilateral tonic-clonic seizures during sleep. By contrast, focal to bilateral tonic-clonic seizures during wakefulness and focal aware seizures were both more common in the SC group than in the UC group. Oral or gestural automatisms were prevalent in UC epilepsy (20.2 vs. 6.9% in the SC group) (*p* = 0.04). Subjective perceptions of seizure onset before loss of awareness (autonomic or psychic) were reported in minority of patients in both groups without statistical differences (*n* = 20, 19.2% in the UC and *n*= 7, 12.1% in the SC group). Ten patients had a single unprovoked seizure, focal to bilateral tonic-clonic during wakefulness in 8 cases, and focal with impaired awareness in the other two.

### EEG Findings

The standard-EEGs (S-EEGs), recorded exclusively during wakefulness, showed IEDs in only 13/104 patients with UC (12.5%), but in 10/58 patients with SC epilepsy (17.2%).

The localization of IEDs was temporal in all the UC group, while SC group also showed parietal and frontal localizations.

The ambulatory-EEG (A-EEG) monitoring (performed in 74.1% of the patients) documented all the NREM stages of sleep, plus Rapid Eye Movement (REM) sleep in all the subjects who underwent this examination. Daytime PSG, documenting sleep until NREM Stage 2, was performed in only 9 patients with SC (5.6% of the cases), the majority of whom (66.7%) showed no IEDs either during wakefulness or during sleep.

These prolonged recording modalities allowed the detection of IEDs in higher proportions of patients (69.2% of the UC group and 55.1% in the SC group). Activation of IEDs during sleep was observed in both groups, with sleep-related IEDs recorded in 72.4% of the UC and 27.5% of the SC group (*p* = 0.02). Also, examination of the sleep portions of the A-EEGs showed that IEDs occurred most frequently in the temporal lobe derivations in the UC group (67.1%), and in the frontal derivations in the patients with SC (65.4%) (*p* = 0.0007).

With regard to the pattern of IEDs across the different sleep stages, the patients with UC showed a higher propensity for spiking during deep NREM sleep (46% of the patients who underwent an A-EEG recording), while the patients with SC during light NREM sleep (*p* = 0.02).

### Neuroimaging Findings

At the time of the diagnosis, all the patients had a neuroradiological examination (CT or MRI) (see Table 1). Taking into account the presence of leukoaraiosis (Fazekas scale score ≥1), the signs of leukoaraiosis were found in both patients with UC and with SC (66.3% of the UC and 79% of the SC cases), without statistical differences between the two groups. Fazekas scale score was significantly lower in UC than in SC patients (median 2 vs. 3, *p* = 0.017).

### Anti-seizure Medications

In both groups, levetiracetam, lamotrigine, and carbamazepine were most commonly the first ASM prescribed, without the statistically significant differences between them; in most cases they were prescribed as monotherapy (79% of cases). A higher proportion of antiseizure polytherapy was found in the SC group (22.1% of cases vs. 18.9% of patients with UC).

The probability of achieving a seizure freedom with the first target dose was significantly different among individual ASM, being higher with levetiracetam and lamotrigine (*p* = 0.005).

### Prognosis

The cumulative probability of achieving seizure freedom at 1, 2, and 3 years, after titration of the first ASM to the first target dose, calculated in the whole sample, were respectively: 68.9% (95% CI 0.621–0.765), 55.6% (95% CI 0.479–0.645), and 45.4% (95% CI 0.375–0.55).

**Figure 1:**
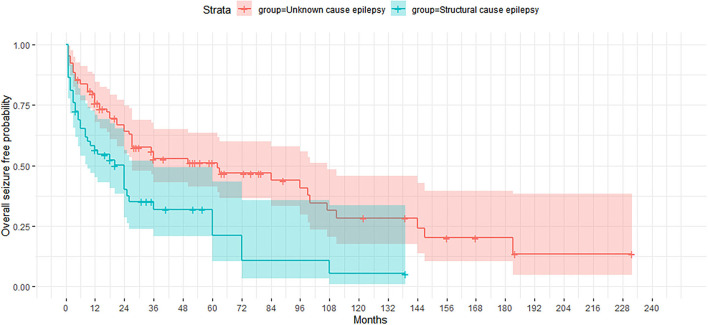
Kaplan-Meier curve showing the proportion of patients who remained seizure-free according to etiology of epilepsy (unknown cause and structural etiology).

On comparing the two groups of patients according to epilepsy etiology (Figure 1), the cumulative probability of seizure freedom was found to be significantly higher in the UC group (*p* < 0.0001).

Considering the seizure characteristics, the absence of subjective perceptions at seizure onset was associated with a higher probability of seizure freedom (*p* = 0.04).

**Figure 2:**
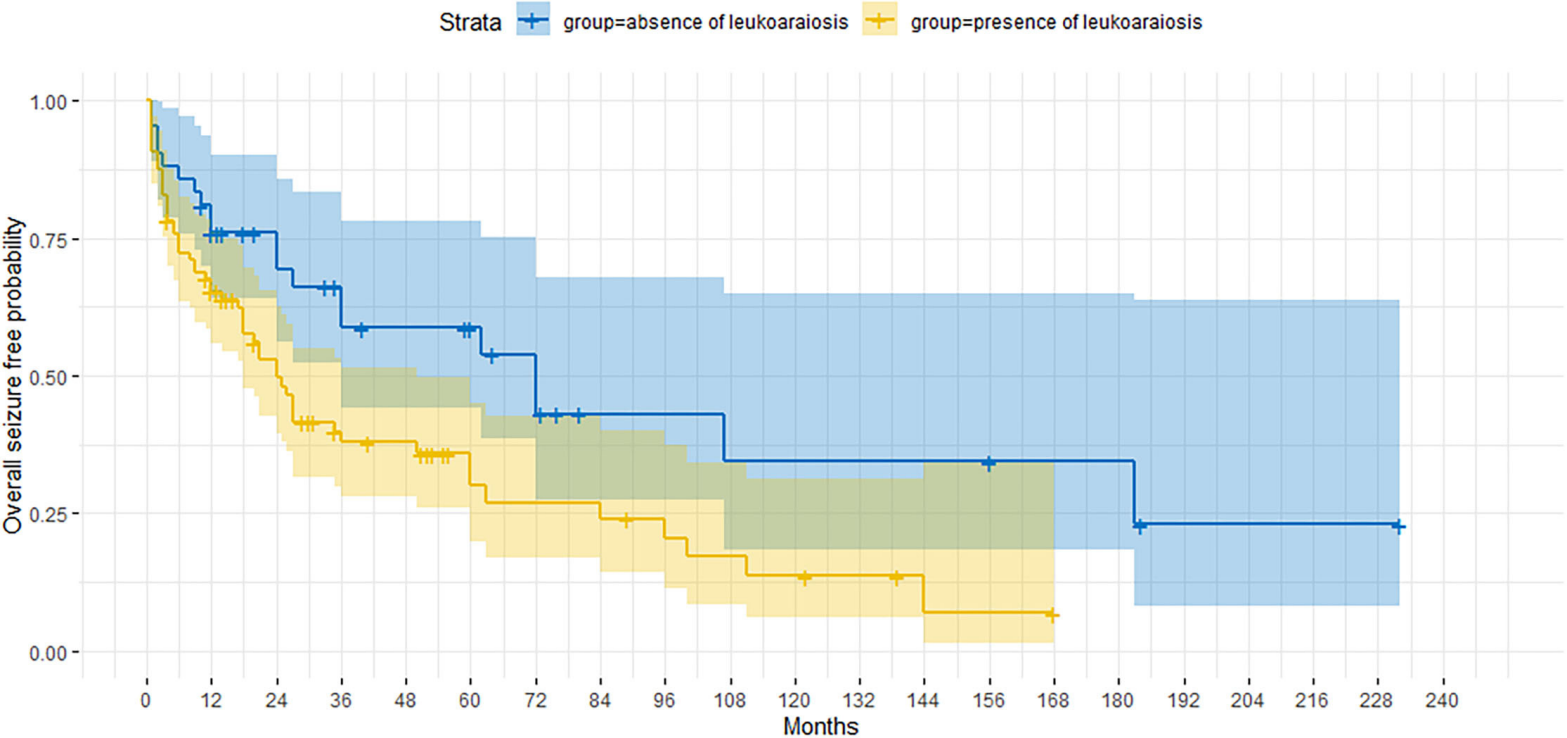
Kaplan-Meier curve showing the proportion of patients who are seizure-free according to the presence/absence of the neuroimaging finding of leukoaraiosis.

The presence/absence of neuroradiological signs of leukoaraiosis significantly affected the cumulative probability of obtaining a seizure freedom, which was significantly higher in the absence of this MRI feature (*p* = 0.012) (Figure 2). When considering the UC group separately, the impact of leukoaraiosis on seizure freedom was similar to that found for the entire cohort (*p* = 0.02). The severity of leukoaraiosis, evaluated through Fazekas scale, did not affect/ the outcome.

The age of the patient at seizure onset and gender did not significantly influence the cumulative probability of seizure freedom.

### Results of the Multivariate Analysis

The following covariates were found to significantly affect the probability of seizure freedom:

- etiology: the patients with UC appeared to have lower risk of seizure recurrence compared with the patients with SC epilepsy (RR=2.243, 95% CI 1.412, 3.561);
- subjective perceptions at seizure onset: the presence of these feelings during seizure appears to be related to a higher risk of seizure recurrence compared with its absence (RR = 2.177, 95% CI 1.298, 3.653).
- Leukoaraiosis: the presence of leukoaraiosis carried a higher risk of seizure recurrence compared with its absence (RR = 1.758, 95% CI 1.012, 3.055).

Table 2 lists the variables included in the Cox regression, and the results of the multivariate statistical analysis.

**Table 2:** Results of multivariate analysis.

**Variable**	**coef**	**z**	**P**	**RR**	**95%CI**
Etiology (SC)	0.79238	3.461	0.0005	2.209	1.410–3.459
Subjective perceptions at seizure onset	0.76954	2.940	0.003	2.159	1.292–3.606
Leukoaraiosis	0.58805	2.109	0.035	1.800	1.042–3.110
Age at onset	−0.01798	−1.104	0.27	0.982	0.951–1.014
Gender	0.16918	0.780	0.44	1.184	0.774–1.812

*SC, structural cause*.

*Concordance:0.643 (se = 0.034)*.

## Discussion

In this study, clinical and EEG differences emerged between the new-onset focal epilepsy outpatients, who were divided into two groups according to the presence or absence of a structural etiology. In particular, in the UC group, epilepsy was characterized by seizures with impaired awareness in wakefulness, and also by a higher proportion of focal to bilateral tonic-clonic seizures during sleep; this profile differed substantially from that of the SC group, which, instead, showed higher proportions of focal to bilateral tonic-clonic seizures in waking hours and focal aware seizures.

### Clinical Features

In elderly patients, focal seizures with impaired awareness have been reported to be the most common seizure type in the community-based studies (1).

With regard to the occurrence of focal to bilateral tonic-clonic seizures during sleep, an Australian hospital-based study analyzing the clinical features and prognosis of first epileptic seizure in a series of elderly subjects, found that healthy older patients were more likely to have a first-ever seizure from sleep (9).

In our series, this propensity seemed to characterize the UC group, and appears to be consistent with previous studies reporting a higher incidence of unknown etiologies in patients with exclusively sleep-related seizures (23–25).

### EEG Features

Our extensive EEG assessment performed in the first referral period, which in most cases included sleep EEG recording, showed a low sensitivity of S-EEG in detecting IEDs, as well as remarkably increased IEDs detection during NREM sleep, in both patients with UC and with SC.

The findings of the present study confirm reports of a low propensity for interictal spiking on S-EEG among elderly outpatients with new-onset and still untreated focal epilepsy, independently of the presence, or otherwise, of a defined etiology (4, 26).

Previously published data refer to a heterogeneous patient series recruited in a variety of settings, often presenting multiple comorbidities and co-medications, including ASM, that could have affected an EEG activity (26, 27).

Some studies, mostly performed in adults with drug-resistant focal epilepsy on ASM treatment, have found that temporal lobe epilepsy shows a greater propensity for interictal spiking during deep NREM sleep (28–31).

In two previous studies, dealing respectively with drug-resistant, mainly symptomatic, temporal lobe epilepsies, and with new-onset, untreated focal epilepsies of unknown cause, aging has been shown to increase this propensity (4, 32).

In the present study, the A-EEG findings in the UC group documented a greater incidence or activation of IEDs during deep NREM sleep, thus, supporting the clinical suggestion that a new-onset epilepsy of unknown cause in otherwise healthy elderly subjects is mainly of temporal lobe origin. Instead, in the SC group, both the clinical and the EEG findings were consistent with extratemporal (mainly frontal lobe) epilepsy.

### Prognosis

The probability of achieving a seizure freedom with the first ASM was high in the sample as a whole. This finding appears to be consistent with previous reports, in which, most of the included patients had symptomatic epilepsy (6, 7, 10, 33).

The factors affecting the prognosis of epilepsy in the different stages of life are still poorly known (34).

Some literature suggests that older age, *per se*. is a predictor of a good seizure outcome, while other authors found this factor to be uninfluential (7, 9, 33).

Among the studies focusing on epilepsy in the elderly, only that of Besocke and colleagues reported a better prognosis in structural epilepsy than in the epilepsy of unknown cause. Also, in elderly subjects, an early response to antiseizure treatment has been found to be a major indicator of a good prognosis (8, 35).

In our study, the absence of a known etiology and the absence of subjective perceptions at seizure onset favorably affected the prognosis, whereas no other clinical variable was found to have a prognostic role.

Nevertheless, in our series, the presence of leukoaraiosis has negatively affected the probability of achieving a seizure freedom, being burdened by a near two-fold increased relative risk of seizure recurrence.

Leukoaraiosis has a complex and not yet clearly understood pathogenesis (36). The age and hypertension are the most relevant risk factors, but also diabetes, smoking, and hyperhomocysteinemia are known to predispose to small vessel disease (37–39). Taking these factors into consideration may be even more relevant in elderly patients treated with enzyme-inducing antiseizure medications, which could increase the plasma homocysteine levels (40, 41).

Notably, hypertension has long been recognized to be independently associated with unprovoked seizures (42–44), both *via* direct mechanisms involving the renin-angiotensin system, and indirectly adding an increased predisposition to small vessel disease (45).

The association of leukoaraiosis with epilepsy has been explored both in experimental studies of epileptogenesis and in clinical investigations (14, 45, 46).

Several case-control studies on neuroimaging in the late-onset epilepsy have provided interesting but conflicting data on the relevance and amount of cerebrovascular disease in these patients (42–44).

Recently, a longitudinal prospective study of a large cohort of patients proved the existence of an independent association between early white matter brain hyperintensity and late-onset epilepsy (47).

In a comparison of clinical characteristics of adult/elderly patients with post-stroke epilepsy and epilepsy associated with the presence of leukoaraiosis, the latter was more frequently associated with a presumed temporal lobe localization of the epileptogenic zone (14).

In this retrospective study, no conclusions can be drawn regarding the possible relationships between the findings linking the UC epilepsy to the temporal lobe and to other variables that are commonly considered in elderly patients, such as neuropsychological profile and advanced imaging characterization. Furthermore, our outpatients, being referrals to a third-level epilepsy center are not necessarily representative of newly diagnosed epilepsy cases in the general elderly population.

Nevertheless, our data are derived from a homogeneous diagnostic protocol, including an extensive electroclinical characterization of patients from a seizure onset, and from prolonged patient follow-ups, carried out exclusively by a single senior epileptologist.

On the basis of the evidence presented here, it appears conceivable that factors, which are seemingly unrelated to the etiology of epilepsy, could influence its clinical expression and prognosis, in ways that further prospective studies might help to elucidate.

## Conclusion

Our study confirmed that epilepsy in the elderly has a good treatment prognosis.

It has also showed that:

- late-onset focal epilepsy of unknown cause is characterized, with respect to symptomatic epilepsy, by the presence of focal seizures with impaired awareness and focal to bilateral tonic-clonic sleep-related seizures, by IEDs localization within the temporal lobe on S-EEG, and by activation of IEDs during the deep stages of NREM sleep, findings that altogether suggest a temporal lobe origin of the seizures;
- late-onset focal epilepsy with unknown cause has a significantly better prognosis than symptomatic forms; and
- the presence of leukoaraiosis on neuroimaging significantly worsens the seizure prognosis as an independent factor.

In accordance with the findings of our study, it seems important, for diagnostic purposes in elderly-onset epilepsy, to exploit EEG with prolonged recordings, also covering the deep stages of NREM sleep.

## Data Availability Statement

The original contributions presented in the study are included in the article/supplementary material, further inquiries can be directed to the corresponding author.

## Ethics Statement

Ethical review and approval was not required for the study on human participants in accordance with the local legislation and institutional requirements. The patients/participants provided their written informed consent to participate in this study.

## Author Contributions

ET: methodology, data collection, writing, review, and editing of the draft. EM and SS: data collection and review of the draft. EB: performed the statistical analysis and review of the draft. MP: data collection, review of the neuroimages, and review of the draft. CG: conceptualization, methodology, writing, review, and editing of the draft. All authors agree to be accountable for the content of the work.

## Conflict of Interest

The authors declare that the research was conducted in the absence of any commercial or financial relationships that could be construed as a potential conflict of interest.

## Publisher's Note

All claims expressed in this article are solely those of the authors and do not necessarily represent those of their affiliated organizations, or those of the publisher, the editors and the reviewers. Any product that may be evaluated in this article, or claim that may be made by its manufacturer, is not guaranteed or endorsed by the publisher.
